# Pareto task inference analysis reveals cellular trade-offs in diffuse large B-Cell lymphoma transcriptomic data

**DOI:** 10.3389/fsysb.2024.1346076

**Published:** 2024-03-01

**Authors:** Jonatan Blais, Julie Jeukens

**Affiliations:** ^1^ Oncology Research Axis, Centre de Recherche du CHU de Québec-Université Laval, Quebec City, QC, Canada; ^2^ Department of Laboratory Medicine, CHU de Québec-Université Laval, Quebec City, QC, Canada

**Keywords:** pareto theory, transcriptomics, lymphoma, oncology, archetypes, trade-offs, optimality, systems biology

## Abstract

One of the main challenges in cancer treatment is the selection of treatment resistant clones which leads to the emergence of resistance to previously efficacious therapies. Identifying vulnerabilities in the form of cellular trade-offs constraining the phenotypic possibility space could allow to avoid the emergence of resistance by simultaneously targeting cellular processes that are involved in different alternative phenotypic strategies linked by trade-offs. The Pareto optimality theory has been proposed as a framework allowing to identify such trade-offs in biological data from its prediction that it would lead to the presence of specific geometrical patterns (polytopes) in, e.g., gene expression space, with vertices representing specialized phenotypes. We tested this approach in diffuse large B-cell lymphoma (DLCBL) transcriptomic data. As predicted, there was highly statistically significant evidence for the data forming a tetrahedron in gene expression space, defining four specialized phenotypes (archetypes). These archetypes were significantly enriched in certain biological functions, and contained genes that formed a pattern of shared and unique elements among archetypes, as expected if trade-offs between essential functions underlie the observed structure. The results can be interpreted as reflecting trade-offs between aerobic energy production and protein synthesis, and between immunotolerant and immune escape strategies. Targeting genes on both sides of these trade-offs simultaneously represent potential promising avenues for therapeutic applications.

## Introduction

Oncology has made tremendous progress over the last few decades (Cancer Progress Timeline | ASCO). New treatment modalities have been developed and existing ones have been refined, combined and optimized to the extent that clinical outcome, including overall survival, has improved significantly for many tumour types (e.g.,(Arnold et al., 2019)). Yet, in many cases, despite these advances, and although response to therapies is often initially good for many types of cancer, resistance develops and leads to relapse/refractory tumours (Vasan et al., 2019). For too many patients, a cancer diagnosis is still synonymous with premature death and cancer still represents one of the main causes of mortality and morbidity in human populations.

With the development of “omics” sciences, the last few decades have also seen the accumulation of a huge amount of publicly available data, especially DNA and RNA sequences, as well as data about numerous signalling pathways and cellular processes. We are therefore facing a situation where the amount of available data is no longer the main limiting factor in advancing cancer therapy. Although, the quality of data in terms of spatio-temporal correlations with respect to relevant pathophysiological processes may still remain out-of-reach of currently available resources and/or technological possibilies. In any case, the availability of such “big data” has yet to propel cancer research to dramatic new therapeutic advances or to cure cancer. Indeed, immunotherapy is probably the most significant innovation in cancer treatment of the last decade and its development does not directly derive from the omics revolution but rather from a more conventional incremental increase in our knowledge of immunology (Paucek et al., 2019).

Arguably, one of the main reasons explaining this inability to take full advantage of the information contained within these public databases stems from the lack of a theoretical framework, organizing principles and methodology allowing to target only their most biologically relevant elements (thus turning data into information). Such a theoretical framework should provide a way to understand the most crucial and essential tasks that malignant cells must perform in order to survive and the genes used to perform these tasks, with the ultimate goal of selectively eliminating malignant cells.

Typically, cancer is understood to be the result of the accumulation of random mutations in genes involved in processes such as cell cycle control, apoptosis and DNA repair, eventually producing the malignant phenotype recognized as cancer (Sonnenschein and Soto, 2020). This view, often called the “somatic”, “reductionist” or “somatic mutation theory (SMT)” does not offer any specific guiding principle to navigate omics data (Selvarajoo and Giuliani, 2023). Under this classical paradigm, basic cancer research aims at providing increasingly detailed descriptions of various genetic alterations and their impact on cellular pathways, but with no reference to a deeper organising theoretical framework to increase our understanding of the phenomenon and thus becomes an essentially descriptive exercise (Monti et al., 2022).

In order to solve what is currently recognized as one of the main problems in cancer therapy, namely, the development of resistance to previously effective treatments (Bukowski et al., 2020; Saleh and Elkord, 2020), an alternative approach might be to try gaining insight into cancer cell vulnerabilities by looking at the fitness of different genotypes through analysis of the genomic fitness landscapes. In principle, this exercise should allow the prediction of cancer evolution under various circumstances. The genomic fitness landscape allows not only the determination of the selection coefficient and gradient associated with any particular allele, but also the identification of critical biological functions in the malignant phenotype and the prediction of response to therapy. In practice however, this is a daunting task as it requires within patient serial measurements at single-cell genomic resolution (Salehi et al., 2021), which is currently out of reach for most research teams.

Interestingly, however, an approach from systems biology may alleviate the need to characterize tumors’ fitness landscapes to identify biologically critical tasks and functions performed by tumor cells. This idea relies on the fact that living organisms and cells need to optimize multiple tasks simultaneously and inevitably face trade-offs in doing so. Mathematically, it can be shown that such constraints imply that the data will form polytopes (n-dimensional generalization of 3-dimensional polyhedra: geometrical object with flat polygonal faces, straight edges and sharp vertices) in trait space (e.g., gene expression space) where vertices of the polytope each represent the optimal specialist phenotype for performing a given task (Hart et al., 2015). Known as Pareto task inference (implemented in the ParTI package in MatLab available at: Pareto Task Inference (ParTI) method | Uri Alon (weizmann.ac.il)), this idea comes from the general Pareto optimality theory that has been used in fields like engineering and economics for many decades in order to solve multi-task optimization problems involving trade-offs between different objectives. Briefly, ParTI allows the use of algorithms to find the best fitting simplex (the simplest polytope) on the data after dimensionality reduction by principal component analysis (PCA), and to test the statistical significance of the simplex by means of the t-ratio test. This test computes the ratio between the area encompassed by the fitted polytope to the area defined by the convex hull of the data (the t-ratio) and then repeat this process after randomizing the data in order to obtain an empirical null distribution for the t-ratio. Pareto task inference has so far been applied to a few biological datasets, including solid tumor gene expression (Hausser et al., 2019; Hausser and Alon, 2020). Most importantly, by circumventing the need to reconstruct the fitness landscape, this method has the potential to directly unveil the most relevant cellular processes for therapeutic intervention by allowing one to infer the fitness consequences of disrupting the cell’s ability to carry out specific tasks.

Although some authors have raised the possibility that the ParTI algorithm might produce spurious results due to phylogenetic, ancestral or other population structure (Sun and Zhang, 2020; Mikami and Iwasaki, 2021), Adler et al. (Adler et al., 2022) tested whether such problems could affect cancer gene expression data and found no evidence that this was the case. The risk of false-positives due to “p-hacking” through data preprocessing has also been raised (Sun and Zhang, 2020). Amid these concerns, rather than speculating on possible artefactual polytopes or on the possibility of statistical false-positives, a more fruitful way of addressing the validity of the ParTI method is to derive specific predictions of the Pareto optimization theory besides the geometrical prediction of polytopes in trait space. In particular, the biological interpretability and underlying logic of the results obtained from that method appear to be of particular relevance in that regard.

For instance, if the most specialized cellular phenotypes (the “archetypes”) identified as the vertices of the polytope in gene expression space are the results of artefactual data structure unrelated to biological specialization and optimization, one would not expect to find statistically significant and distinct enrichment in particular biological functions at these archetypes. Moreover, if polytope formation results from optimization trade-offs, as per the theory, the various tasks defining the archetypes should all be considered important, if not essential, for cell survival. Indeed, trade-offs imply that some of these tasks cannot be simply eliminated completely from the cell’s functional repertoire, otherwise there should be no trade-offs constraining the optimization process. Hence, archetypes represent different solutions of proportional allocations of resources to these essential functions. It is therefore likely that different archetypes would show different proportions of a number of shared functions/genes, reflecting these different solutions. Alternatively, a pattern where all vertices are characterized by a unique and distinct set of functions and gene-transcripts would not be easely interpretable as the results of trade-offs constraining the cell’s ability to maximize certain functions to their full genetic potential since each vertex would represent a unique and distinct functional domain. Thus, if the existence of the archetypes identified by the ParTI algorithm is the results of optimization trade-offs as predicted by the Pareto optimization theory, we should expect these archetypes to be statistically enriched in specific biological functions, and to be characterized by varying proportions of some shared tasks/genes in different combinations.

Because of its potential as a useful theoretical principle allowing meaningful analysis of omics and other biological data, and thus to provide important information about both basic biology and therapeutically actionable knowledge, the Pareto task inference approach should be carefully and independently evaluated and validated. So far, the ParTI package has been used on a few solid tumor datasets but, to our knowledge, never on hematological malignancies. Because of their cell type (malignant lymphocytes) and phylogenetic (from clonal proliferation) high level of homogeneity, data from lymph node biopsies of lymphoma patients are particularly well suited to study the validity of this method. In order to test the above predictions, we analyzed transcriptomic data from diffuse large B-cell lymphoma (DLBCL) samples for 481 patients using ParTI code. Archetypes identified by the algorithm were then tested for functional enrichment and compared in terms of gene composition and representation. Results were ultimately evaluated for their interpretability, potential biological meaning and usefulness for generating hypotheses.

## Materials and methods

### Gene expression data

Data were obtained through the National Cancer Institute’s Genome Data Commons Data Portal (GDC (cancer.gov)). Gene expression quantification expressed as transcripts per millions (TPM) from RNASeq was available for 517 DLBCL patient-lymph node biopsy samples (cases.disease_type in ["mature b-cell lymphomas"] and cases. primary_site in ["lymph nodes"]) from projects NCICCR-DLBCL, TCGA-DLBC, and CTSP-DLBCL1. Of these, 481 (93%) were from the NCICCR-DLBCL project, while only 29 (5.6%) and 7 (1.4%) were from the TCGA-DLBC and CTSP-DLBCL1 projects respectively. In order to ensure maximum homogeneity in sampling and sequencing protocol and hence between-sample comparability of data (Zhao et al., 2020), we analyzed the 481 cases from the NCICCR-DLBCL project. All 481 samples were fresh frozen lymph node biopsy samples, 96.3% collected prior to any treatment. The same RNASeq protocol was applied to all samples (Schmitz et al., 2018). We kept all informative genes/isoforms (22 250), defined as having standard deviation and variance ≥1 TPM, for downstream analyses. To avoid p-hacking issues, we refrained from performing any preprocessing transformations/manipulations of data and used the raw TPM gene expression quantification. TPM is considered a better unit of RNA abundance than RPKM/FPKM since it respects the invariant-average property (the average TPM is a constant equal to 10^6^ divided by the number of annotated transcripts) and is proportional to the average RNA molar concentration (rmc). It has thus been adopted by the latest computational algorithms for transcript quantification such as RSEM, Kallisto and Salmon (Zhao et al., 2020). The resulting data matrix had 481 sample lines by 22 250 genes/isoforms columns.

### PCA and pareto task inference analyses

Because the ParTI code package relies on principal component analysis (PCA) for dimensionality reduction, as proposed by Vieira (Vieira, 2012), we first tested the validity of performing PCA on the data by computing the *Ψ* and *ϕ* statistics, the number of significant principal components, as well as the number of genes/isoforms with significant correlations with each of the principal components, by permutations and bootstraping using PCAtest in R (Camargo, 2022).

Following Mikami and Iwasaki (Mikami and Iwasaki, 2021), the simplex best fitting the data was determined by the SDVMM algorithm (Chan et al., 2012b). Five such fitting algorithms are available in ParTI. The SISAL algorithm is not recommended for datasets of less than about 1000 data points (or more precisely of less than $10^{N}$ points, with *N* being the number of PCA dimensions), and will estimate the archetypes outside of the convex hull of the data, while potentially leaving important points outside of the fitted polytope and t-ratio test analysis (PartiCode homepage: Pareto Task Inference (ParTI) method | Uri Alon (weizmann.ac.il) accessed 11/03/2023), which may generate false positive or false negative results of the t-ratio test. The MVA and MVE algorithms, which also locate the archetypes outside of the convex hull, could be used with smaller datasets of <1000 points, but are not robust to noise and outliers. Contrary to the previous algorithms, the PCHA algorithm estimate the archetypes within the data but also suffer from a susceptibility to noise and outliers. Thus, we agree with Mikami and Iwasaki (Mikami and Iwasaki, 2021) that the SDVMM algorithm is the only option combining a strict data constraint with a robustness to noise and outliers and seems clearly preferable for statistical testing. The default value of eight PCA dimensions, as per the ParTI’s example file “exampleCancerRNAseq.m”, was kept as input. Using ParTI’s discrete and continuous attribute functions, we also tested whether archetypes were associated with three clinical correlates available in the dataset, namely, tumor cell of origin (COO) subtype (which was available for all samples), progression free survival (PFS) in years (which was available for 48% of the samples), and the international prognostic index (IPI) score (which was available for 74% of the samples).

Archetype coefficients in original gene expression space were then extracted from ParTI’s output and genes ordered according to coefficient value. Genes with the highest positive and negative coefficient values for each archetype (genes with the highest PCA loadings at the archetype location and thus defining the archetype in terms of most positively/negatively correlated expression values) were identified by plotting ordered coefficient values and visually identifying the abrupt change in slope between the vast majority of genes with coefficients close to zero and the few genes with highly positive (highly positively correlated) or negative (highly negatively correlated) values, and taking this inflexion point as cut-off. These archetype-defining gene lists were then tested for functional enrichment using Gene Ontology tools (GO aspect: biological process) (Ashburner et al., 2000; Thomas et al., 2022; Aleksander et al., 2023). Functions with a Fisher’s exact test enrichment *p*-value <0.05 (after adjustment for false-discovery rate (FDR)) were considered significantly enriched at the archetype. The composition of significantly enriched functions was compared and the level of shared vs. unique functions among archetype analyzed using Circos (Krzywinski et al., 2009).

### Data availability

The dataset analysed for this study is publicly available on the Genome Data Commons Data Portal at GDC (cancer.gov). The code needed to replicate the ParTI analysis from these data is provided as Supplementary Table S1.

## Results

### PCA validation

PCAtest was run for 100 permutations and bootstrapping replicates and estimated an empirical *Ψ* value of 51848193.065, with a max null *Ψ* = 979604.238 and a min null *Ψ* = 978848.881, for a *p*-value <0.00001. Likewise, the *ϕ* value was estimated at 0.337, with a max null *ϕ* = 0.047 and min null *ϕ* = 0.047, for a *p*-value <0.00001. These results indicate highly significant non-random correlations in the data, justifying the use of PCA and confirming that the analysis is biologically meaningful. Moreover, the analysis showed that the first eight principal components (PC) explained 80% of the total original variance (from 1.8% to 33% for individual PC). Finally, the number of genes with significant correlations with each of these eight principal components ranged from 2830 to 7546. Together, these results strongly support the use of PCA on this dataset.

### Polytope fitting

**FIGURE 1:**
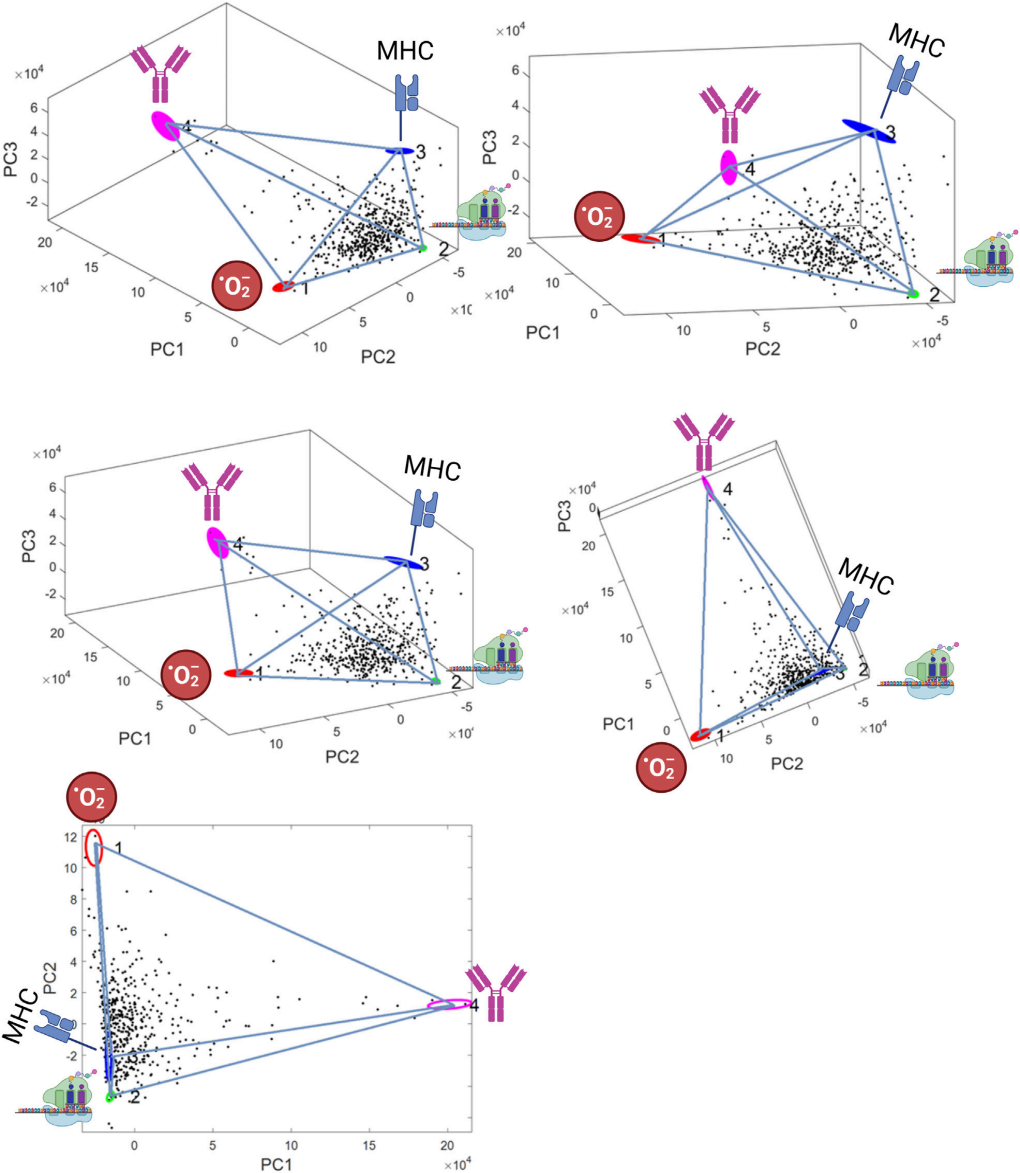
Best fitting polytope identified by the ParTI algorithm. 3-D representations A)-D), and 2-D representation E) are shown in PCA space (the axes are the first three principal components, which explain 66% of variance). The tetrahedron defines four archetypes (coloured ellipses): 1) aerobic energy production (0_2_Energy) 2) protein synthesis, 3) immunotolerant tumor micro-environment (Immunotolerant-TME), 4) “Plasmablast-like” differentiation. The four archetypes are represented by pictograms. Ellipse area is proportional to archetype location uncertainty from 1000 bootstrap replicats.

The elbow method applied by the PartiCode package suggested the presence of four archetypes, thus defining a tetrahedron (3-d simplex) (Figure 1). The identified polytope was highly significant with the t-ratio test indicating a *p*-value <0.00001. The four vertices represented archetypes of 1) aerobic energy production (O_2_Energy), 2) protein synthesis, 3) Immunotolerant tumor micro-environment (Immunotolerant-TME), and 4) Immune-escape via plasmablast-like differentiation (“Plasmablast-like”). A detailed description of the functional analysis characterizing these archetypes follows.

### Archetype-defining genes selection

For each of the four archetypes, ordered coefficients were plotted and revealed a clear pattern of abrupt change from coefficients close to zero for the vast majority of genes, to a few genes showing strongly positive or negative coefficients (Supplementary Figure S1). Archetype-defining genes were therefore selected by considering a horizontal line (slope = 0) for genes with a coefficient close to zero and a line with a slope of 1 (vertical) for genes with strongly positive or negative coefficients. These lines were extrapolated and the line bisecting these right-angled extrapolated horizontal and vertical lines at 45° was determined. The mid-point of this 45-degree line, when positioned so it just touched the data, was considered as the inflexion point (elbow point) and used as the coefficient cut-off to select the positive and negative archetype defining genes (Supplementary Figure S2).

### Enrichment analysis

The above procedure identified between 23 and 132 archetype-defining genes with positive loadings, and between 12 and 85 archetype-defining genes with negative loadings depending on the archetype (Supplementary Table S2). For each archetype, both positive and negative gene lists were submitted to the Panther database for enrichment analysis. All eight archetype-defining gene lists (one positive and one negative loading list for each of the four archetypes) were significantly enriched (after FDR adjustment) in some functions. The 10% functions with the most significant enrichment *p*-value for each of the eight lists are presented in Supplementary Table S3. GO terms associated with each individual archetype-defining genes are listed in Supplementary Datasheet S1.

### Archetype characterization

**FIGURE 2:**
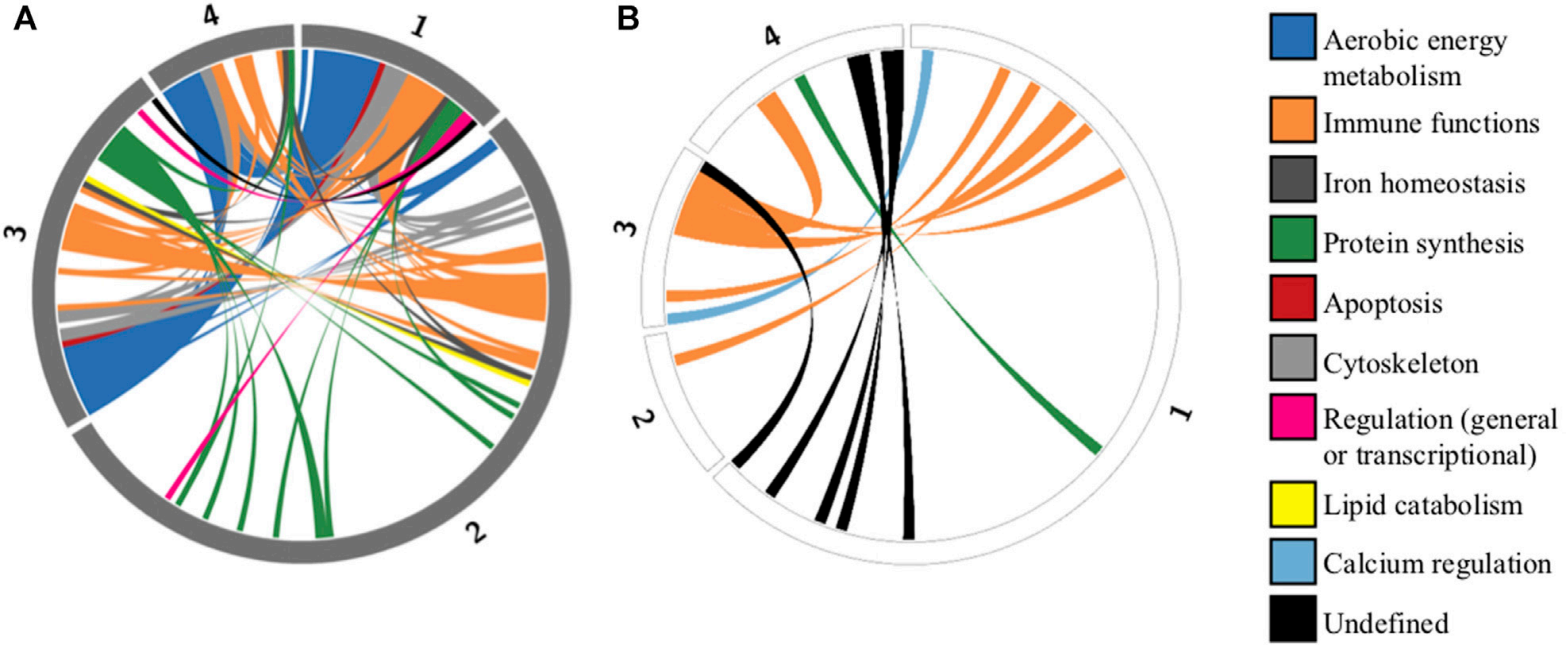
Circos plots representing shared defining genes among archetypes identified by the ParTI analysis. Circular representation of genes defining each archetype. Each segment of the circle represents one archetype (numbered from 1 to 4), and segment length is proportional to the number of archetype-defining genes. Grey segments represent positively correlated genes **(A)**, and white segments represent negatively correlated genes **(B)**. Of the 218 unique defining genes identified in at least one archetype, 107 (49%) are shared between at least two archetypes. These shared genes are connected by colored ribbons, with colors corresponding to functional categories (Supplementary Table S3).

The first archetype was positively enriched in aerobic energy production (three most significant GO terms: oxidative phosphorylation/aerobic respiration/aerobic electron transport chain) and negatively enriched in protein translation (three most significant GO terms: cytoplasmic translation/translation/peptide biosynthetic process). The second archetype was positively enriched in protein translation (three most significant GO terms: cytoplasmic translation/translation/peptide biosynthetic process) and negatively enriched in energy production and immune functions (three most significant GO terms: oxidative phosphorylation/adaptive immune response/cellular respiration). The third and fourth archetype were positively enriched in a mixture of aerobic energy production and immune functions, and negatively enriched in immune functions. The 10% lowest FDR-adjusted *p*-values of the enrichment analysis were very small, ranging from 0.0025 to 5.67 × 10^−95^, indicating that, from a functional point of view, archetype-defining genes represented highly non-random genomic sub-samples. As predicted, a significant proportion (49%) of archetype-defining genes were shared between at least two archetypes, albeit in different combinations and proportions (Figure 2).

Archetype 2 and 3 were significantly associated with the COO “germinal center B-cell” (GCB) and “unclassified” subtypes respectively (*p*-value <0.001 and <0.01; hypergeometric distribution), and those associations remained significant after the Benjamini–Hochberg correction for multiple tests. Archetype 3 was also significantly associated with the IPI “intermediate” score (*p*-value = 0.031; hypergeometric distribution), although the *p*-value did not remain significant after Benjamini–Hochberg correction. Likewise, archetype 3 was significantly associated with a shorter PFS (median difference = −3.2 years; *p*-value = 0.018; Mann -Whitney), while archetype 2 and 4 were marginally associated with a longer PFS (median difference = 3.1 and 3.8 years; *p*-value = 0.056 and 0.085; Mann-Whitney respectively) but these associations became non-significant after Benjamini–Hochberg correction. As mentioned previously, the sample sizes and (therefore statistical power) for testing archetypes association with IPI and PFS were reduced by 26% and 52% respectively.

### Biological interpretability

#### Archetype 1 and 2: metabolic trade-offs between aerobic energy production vs. protein synthesis

**FIGURE 3:**
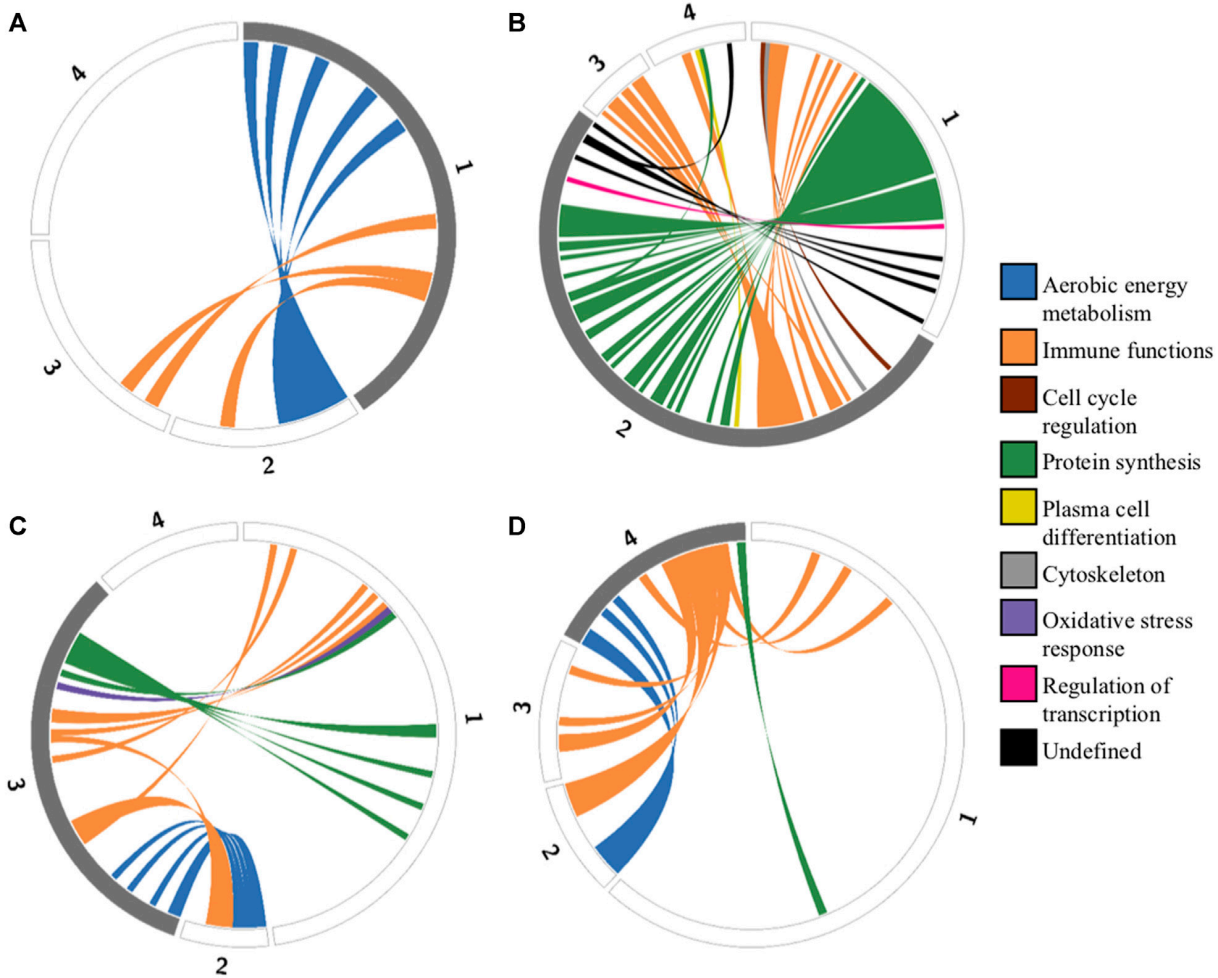
Circos plots representing functional tradeoffs among archetypes identified by the ParTI analysis. Circular representation of genes defining each archetype. Each segment of the circle represents one archetype (numbered from 1 to 4), and segment length is proportional to the number of archetype-defining genes. Grey segments represent positively correlated genes, and white segments represent negatively correlated genes. Genes that are positively correlated in one archetype **(A)** archetype 1, **(B)** archetype 2, **(C)** archetype 3, **(D)** archetype 4) and negatively correlated in another are connected by colored ribbons to emphasize tradeoffs. These colors correspond to functional categories (Supplementary Table S3).

One of the most striking features of the functional enrichment analysis was the contrast between archetype 1, which showed specialization for oxidative phosphorylation (OXPHOS) at the expense of protein synthesis, and archetype 2 which was specialized in protein synthesis at the expense of both OXPHOS and immune functions (Figures 3A, B). A detailed analysis of the genes with expression positively correlated with archetype 2 and negatively correlated with archetype 1 revealed 34 genes involved in protein synthesis. These genes consisted of various ribosomal subunits and two translation elongation factors, as well as CHCHD2. Interestingly, it was recently shown that in stress conditions produced by carbonyl cyanide m-chlorophenylhydrazone (CCCP) treatment, a decoupling agent known to induce oxidative stress (Park et al., 2015), CHCHD2 knockdown triggered the integrative stress response (ISR) in cultured HeLa cells (Ruan et al., 2022). The role of the ISR is to maintain homeostasis under stress conditions, including oxidative stress, and is known to slow down protein synthesis via the phosphorylation of eIF2α (Bilen et al., 2022). Indeed, the ISR was recently observed to inhibit protein synthesis in the context of oxidative stress from mitochondria-derived production of reactive oxygen species (ROS) in a cardiac ischemia/reperfusion model (Zhang et al., 2021). Because of the high rate of energy production via OXPHOS and the associated ROS generated, archetype 1 cells may downregulate CHCHD2 in order to restore homeostasis via the ISR, at the expense of protein synthesis. In contrast, archetype 2 cells may overexpress CHCHD2 in order to benefit from its anti-apoptotic effect (Jiang et al., 2022). Recently, the importance of CHCHD2 in cancer and its potential as drug target was highlighted by Gundamaraju et al. (Gundamaraju et al., 2020). Consistent with the hypothesis of a driving role for increased ROS production in archetype 1, the expression of ROMO1 was negatively correlated with this archetype. ROMO1 is known to increase ROS production (Amini et al., 2019) and thus might be downregulated by archetype 1 as an adaptation to compensate for the high ROS generated by energy production via the electron transport chain.

Another noticeable contrast between archetypes 1 and 2 was the positive correlation of the expression of CKS2 and TPT1 with archetype 2, while the expression of these two genes were negatively correlated with archetype 1. CKS2 and TPT1 have both been implicated in cancer, including as potential therapeutic targets (Acunzo et al., 2014; You et al., 2015; Lee et al., 2022). TPT1 encodes the translationally controlled tumor protein (TCTP) and CKS2 encodes the cyclin-dependent kinase regulatory subunit 2, which is known to bind to and be necessary for the activity of the cyclin B1-CDK1 protein kinase, an essential factor for cells to progress past the G2 phase of the cell cycle (Wang et al., 2021). Interestingly, both TPT1 and CKS2 have also been associated with OXPHOS-induced oxidative stress (Lucibello et al., 2011; Jonsson et al., 2019), as well as with cell-cycle regulation and with protein synthesis/degradation (Bommer and Telerman, 2020; Grey et al., 2023). In an hematopoietic cells (HSC) mouse model, CKS2 knockout was associated with an accelerated cell cycle (Grey et al., 2018) (which may contribute to the malignant phenotype) but also with an increase ROS production (Grey et al., 2023). Moreover, the same study also found that CKS2 was involved in proteostasis of HSC, which may be related to the trade-off in protein synthesis identified for archetype 1 (Grey et al., 2023). The role of TCTP in protein synthesis regulation is known to involve its interaction with elongation factors eEF1A and eEF1B (Bommer and Telerman, 2020). The fact that both eEF1A1 and eEF1B2 followed the same correlation of expression patterns between archetypes 1 and 2 as TPT1 and CKS2 supports this interpretation.

In general, TPT1/TCTP is thought to protect cells against apoptosis and oxidative stress (Bommer, 2017). It might thus seem surprising that its expression should be negatively correlated with archetype 1 given the proposed trade-offs involving increased OXPHOS-induced ROS and ISR activation. However, while mild oxidative stress was found to upregulate TCTP, strong oxidative stress was found to downregulate its expression (Lucibello et al., 2011), and both CKS2 and TPT1 were downregulated in a butyrate resistant cell line conferring tumorigenesis, apoptosis and stress resistance in a colon adenocarcinoma model (López de Silanes et al., 2004). Thus, the level of oxidative stress in archetype 1 might reach levels associated with reduced TCTP expression.

TCTP and CKS2 expression patterns are linked through the TCTP/CDC25C/CDK1 pathway, which was shown to be dysregulated in hepatocellular carcinoma (Chan et al., 2012a). Overexpression of TPT1 has been associated with reduced CDK1 activity via ubiquitin-proteasome degradation of CDC25C, which is necessary for the dephosphorylation and activation of CDK1 (Chan et al., 2012a). By contrast, CKS2 is thought to promote CDK1 expression (You et al., 2015) and to be required for its function. Thus, the reciprocal correlation patterns seen between archetype 1 and 2 might reflect the need to compensate for the reduced activation of CDK1 by CDC25C from the increased expression of TPT1 by an increase in CKS2 expression and *vice versa*.

**FIGURE 4:**
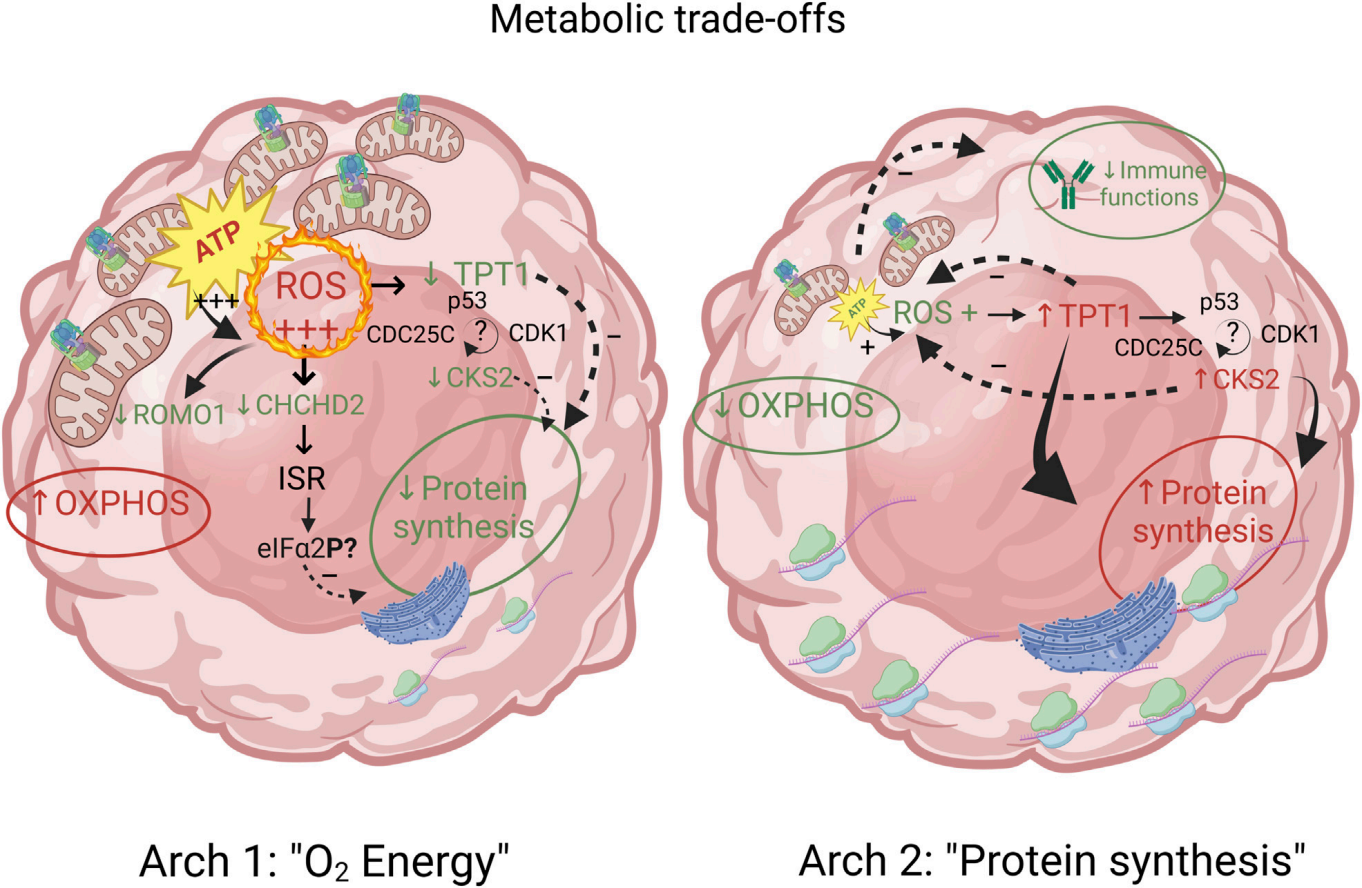
Metabolic trade-offs between archetypes 1 and 2 are characterized by genes involved in energy production, protein synthesis and immune functions. The proposed model of mechanistic relations underlying these trade-offs between aerobic energy production and protein synthesis involves a complex interplay between ROS production and its inhibitory effect on protein synthesis, and minimal energy requirement for protein synthesis and immune functions. Archetype defining functions and genes are shown in red (positively correlated) and green (negatively correlated). Functions and genes in black are hypothetically inferred and not part of archetype defining genes.

**FIGURE 5:**
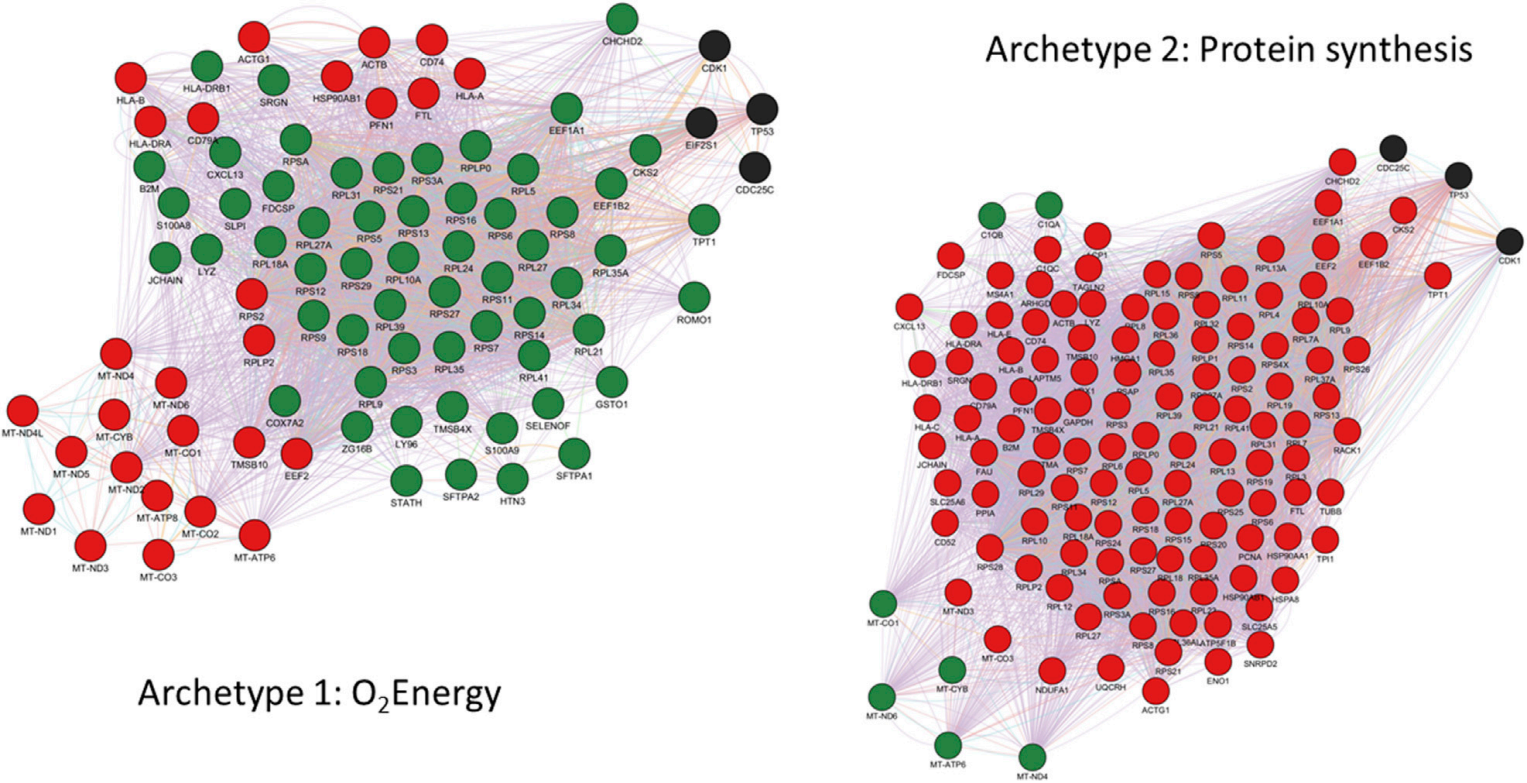
GeneMANIA functional interactions network among genes defining archetype 1 and 2. Networks include co-expression, predicted interactions from orthologs, physical interactions, shared pathway, co-localization, genetic interactions and shared protein domains. Genes with positive and negative expression correlations are shown as red and green nodes respectively. Black nodes represent putatively involved genes inferred from the mechanistic model summarized in Figure 4. Immunoglobulin genes are not included in GeneMANIA searches and are therefore absent from the network. However, JCHAIN is included and can be interpreted as proxy for (pentameric) IgM and (dimeric) IgA expression.

In addition, TCTP and CKS2 were both found to exhibit reciprocal repression with p53. In the case of TPT1, p53 is repressed via TCTP ubiquitin-mediated degradation of p53 while p53 directly represses TPT1 transcription (Amson et al., 2012; Acunzo et al., 2014). Likewise, CKS2 expression was found to be repressed by p53 (Rother et al., 2007), while the overexpression of CKS2 was associated with reduced p53 protein abundance in gastric cancer (Kang et al., 2009). However, the mechanism by which TPT1 and CKS2/CDK1 are repressed by p53 has been questioned and might be due to the indirect DREAM pathway rather than direct interaction with p53 (Fischer et al., 2014). Interestingly, CDC25C was also found to be repressed by p53 (Liu et al., 2020) and thus complex TPT1/CDC25C/CKS2/CDK1/p53 interactions might be behind the TPT1-CKS2 opposite correlation pattern seen in these two archetypes. A schematic and synthetic representation of the hypothetical model outlined above for the metabolic trade-offs suggested by the analysis of archetypes 1 and 2’s defining genes is provided on Figure 4. The full network of functional interactions among genes characterizing archetype 1 and 2 was created using the GeneMANIA app in Cytoscape v.3.10.1 (Montojo et al., 2010) and is shown on Figure 5.

#### Archetype 3 and 4: immune evasion trade-offs between immune-tolerant tumor micro-environment vs. immune escape via plasmablast-like differentiation

A breakdown of the genes characterizing archetype 3 and 4 was also conducted (Figures 3C, D). Many of these genes turned out to be known for their involvement in cancer in general and/or lymphoma in particular. For instance, LMO2 expression was negatively correlated with archetype 4, while IGHM expression was positively correlated with this archetype. This expression pattern has previously been associated with the activated B-cell (ABC) DLBCL COO subtype (Blenk et al., 2007). LMO2 expression reduces double-strand break DNA repair mechanisms and has been associated with a better prognosis in DLBCL patients treated with poly(ADP-ribose) polymerase (PARP) inhibitors (Parvin et al., 2019). Interestingly, the expression of HLA-A,B,C,E, B2M and HLA-DRA/DRB1 was positively correlated with archetype 3, while expression of immunoglobulins (Ig) IGHM, IGHV4-34, IGHV5-51, IGKV3-20, IGLC2, IGLV1-47, IGLV3-1, IGLV3-19, IGLV3-21, JCHAIN were negatively correlated. In contrast, the expression of several Ig genes was positively correlated with archetype 4, while only the invariant HLA-DRA was positively correlated with the archetype. This pattern would be consistent with an immune system escape from MHC loss (de Charette and Houot, 2018) via a partial plasmablast cell differentiation pathway (Wilkinson et al., 2012) in archetype 4. Such a strategy would also be consistent with the many pro-tumorigenesis effect of cancer derived Ig that have been identified, including proliferation, migration, invasion, survival, and immune evasion through inhibitory effect on antibody-dependent cell-cytotoxicity (ADCC) from NK cells (Cui et al., 2021). This picture is also consistent with archetype 4 resembling the ABC subtype of the COO classification (Wilkinson et al., 2012; van der Meeren et al., 2018; Takahara et al., 2023).

**FIGURE 6:**
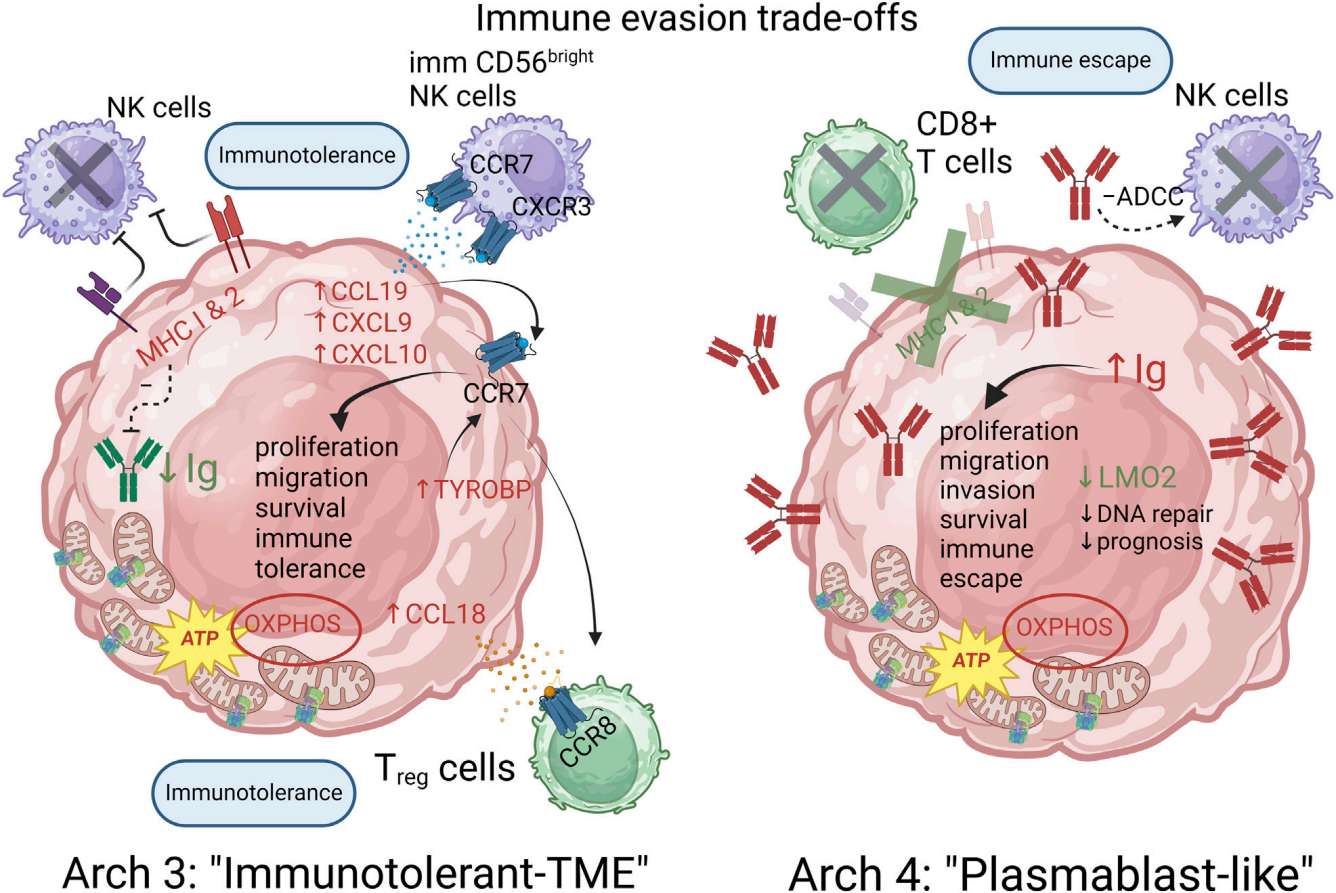
Immune evasion trade-offs between archetypes 3 and 4 are characterized by an immunotolerant and plasmablast-like differentiation strategies respectively. The proposed model posits that MHC expression provides a way to avoid NK cells cytotoxicity while the expression of CCL18/19 and CXCL9/10 promotes an immunotolerant TME by recruiting immature and indolent CD56 “bright” NK cells as well as Treg cells in the case of archetype 3. Archetype 4 achieves an alternative escape from the immune system by downregulating MHC expression, thereby avoiding CD8^+^ T cells cytotoxicity and inhibiting NK cells action via ADCC inhibiting action of certain cancer Ig. Archetype defining functions and genes are shown in red (positively correlated) and green (negatively correlated). Functions and genes in black are hypothetically inferred and not part of the archetype defining genes.

**FIGURE 7:**
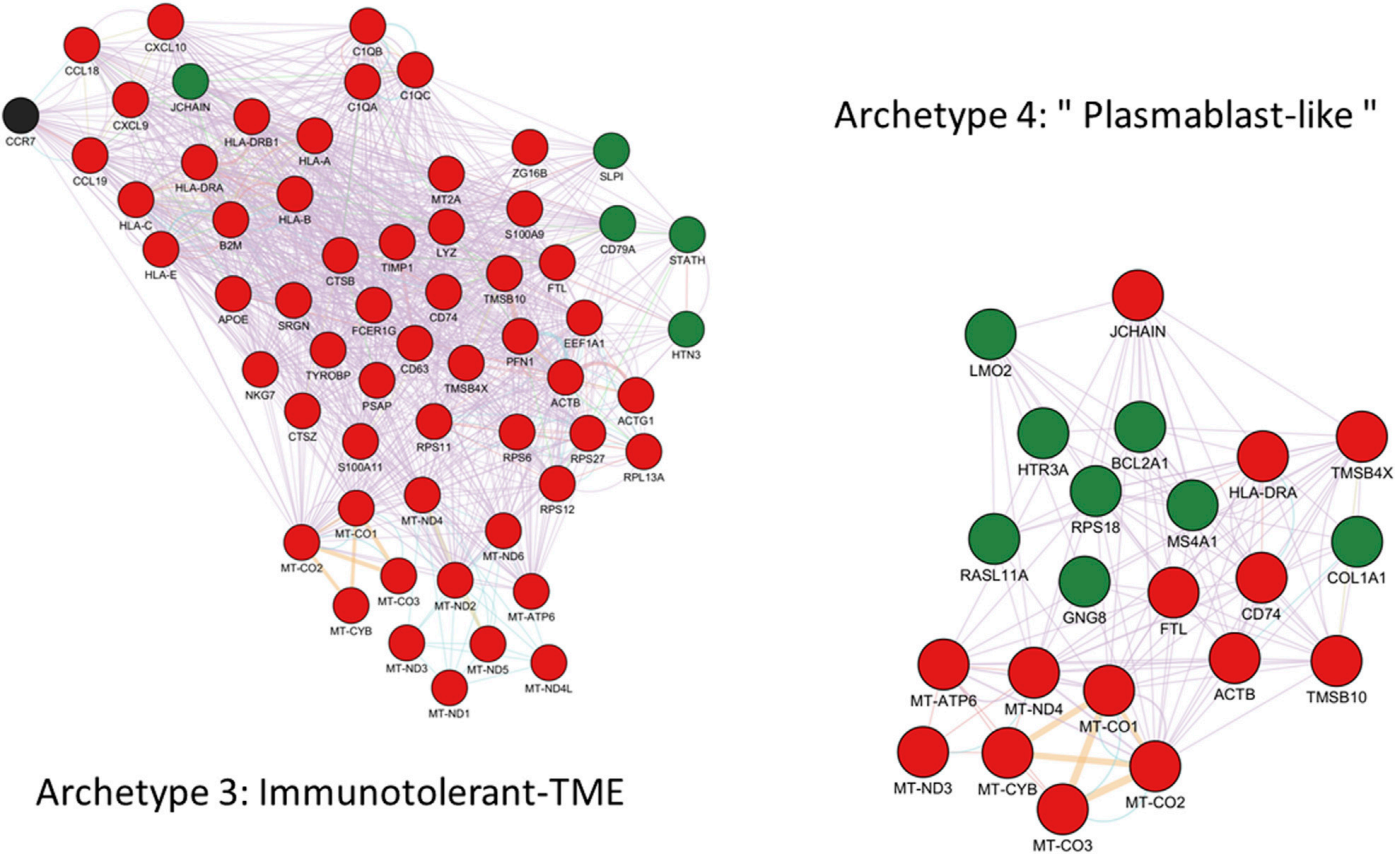
GeneMANIA functional interactions network among genes defining archetype 3 and 4. Networks include co-expression, predicted interactions from orthologs, physical interactions, shared pathway, genetic interactions and shared protein domains. Genes with positive and negative expression correlations are shown as red and green nodes respectively. Black nodes represent putatively involved genes inferred from the mechanistic model summarized in Figure 4. Immunoglobulin genes are not included in GeneMANIA searches and are therefore absent from the network. However, JCHAIN is included and can be interpreted as proxy for (pentameric) IgM and (dimeric) IgA expression.

However, since Ig are common neo-antigens in B-cell malignancies and Ig-derived neoantigen presentation by MHC is a general phenomenon in lymphomas including DLBCL (Khodadoust et al., 2019), archetype 3 may limit the production of Ig in the context of retained MHC expression to avoid displaying neo-antigen Ig to the immune system (Han et al., 2022a). Moreover, archetype 3 was also positively correlated with the expression of CCL18, CCL19, CXCL9 and CXCL10. CCL18 is known to increase the proliferation of B-cell lymphoma (Korbecki et al., 2020) and may contribute to immune evasion from its effect on immune surveillance mediated by macrophages and dendritic cells and by simultaneously favoring T cell-tolerance (Korbecki et al., 2020; Cardoso et al., 2021; Kidani et al., 2022). CCL19 directs B-cell migration after activation via antigen binding and is known to be upregulated in both GCB and ABC DLBCL subtypes. Recently, autocrine CCR7-CCL19 signaling was proposed to significantly contribute to lymphomagenesis under malignant conditions via a stronger activation of the survival pathways (Uhl et al., 2022). More generally, CCR7 signalling upon binding to its ligands (CCL19/21) is associated with many pro-tumorigenic effects in hematological malignancies, including migration, proliferation, survival and immune evasion (Cuesta-Mateos et al., 2021). Interestingly, increased expression of CCL19, CXCL9 and CXCL10 is associated with recruitment of immature CD56^bright^ NK cells with low perforin content in the tumor microenvironment (TME), which is thought to protect tumor cells from NK cells (Castriconi et al., 2018). Additionally, MHC expression is a well-known way for tumor cells to avoid immune detection and destruction by NK cells (Castriconi et al., 2018). This correlation pattern involving MHC/CXCL9-10/CCL18-19 might thus represent the signature of an alternative immune evasion strategy for archetype 3, distinct form the one displayed by archetype 4. Finally, both archetypes were positively correlated with the expression of CD74, a gene involved in B-cell differentiation, proliferation and survival (Zhao et al., 2019). An integrative schematic summary of the immune evasion trade-offs suggested by the above analysis of archetypes 3 and 4 is provided on Figure 6. The full network of functional interactions among genes characterizing archetype 3 and 4 is shown on Figure 7.

### Comparisons with differential expression studies

By looking at some of the genes defining each archetype, trade-offs and expression patterns noted by other studies using different approaches could be detected. Besides the cases already highlighted, the proportion of archetype-defining genes found in nine comparative DLBCL or non-Hodgkin lymphoma gene expression studies (Monti et al., 2005; Dybkær et al., 2015; Michaelsen et al., 2018; Davies et al., 2019; Tripodo et al., 2020; Kotlov et al., 2021; Steen et al., 2021; de Groot et al., 2022; Rapier-Sharman et al., 2022) was assessed. Overall, 23% of the archetype defining genes identified by ParTI were part of the significantly differently expressed genes reported by these studies. Of note, the recently comparative transcriptomic study of Rapier-Sharman et al. (Rapier-Sharman et al., 2022) compared RNASeq gene expression data from 322 samples, including 134 B-cell lymphoma samples (of which 123 were LBCL/DLBCL) and 188 healthy B-cell controls. Among the 20 most differently expressed genes between B-lymphoma and normal control samples, 7 (35%) were found among the archetype defining genes identified here by the ParTI algorithm (CXCL9, CXCL13, C1QA, C1QB, C1QC, CCL18, CCL19). An additional three (15%) archetype defining genes identified by ParTI were among the 20 genes showing the most significant differences in the presence of splice variants (APOE, COL1A1 and RPL5). Although the tasks and trade-offs identified by ParTI are not necessarily expected to match differently expressed genes between malignant and normal cells, the fact that a significant proportion of genes showing differential expression between normal and lymphoma cells in differential expression studies are also found among archetype-defining genes identified by the ParTI approach provides additional and convincing evidence that the identified archetypes are related to the malignant process and phenotype. As such, it increases confidence that the Pareto optimality theoretical framework is uncovering biologically meaningful and interpretable information at the scale of systems organization, with potential therapeutic relevance.

## Discussion

Uncovering cancer cell vulnerabilities in the form of trade-offs represents a promising avenue to avoid the emergence of treatment resistance. The presence of trade-offs implies that certain tasks cannot be completely avoided by the cells, and yet cannot be simultaneously optimized to the maximum level allowable in principle by the genetic potential. The genes underlying these trade-offs are thus attractive therapeutic targets that could make resistance more difficult to acquire for the malignant cells. The Pareto task inference approach predicts that whenever such trade-offs exist, they should produce detectable geometrical structures in the data. We further predicted that besides geometrical patterns in trait space, if the genes located at the vertices of the polytope identified by the algorithm do indeed represent phenotypic optima (archetypes), as predicted by the theory, rather than artefacts unrelated to phenotypic optimization, these genes should be significantly enriched in biological functions and characterized by patterns of different combinations of a certain proportion of shared tasks/genes.

The data analyzed here do indeed confirm those three predictions at a high level of statistical significance. The t-ratio test empirically calculates the probability of observing a polytope providing as good or better fit to the data as compared to the best possible fit defined as the convex hull. To this end, the data are randomized and the best fitting polytope and its ratio to the convex hull for each replicate data set are re-estimated. The value of the observed ratio is then compared to the distribution of ratios from the simulated randomized data to derive its probability. This test strongly supports the presence of a polytope defined by 4 vertices (tetrahedron) in this transcriptomic dataset.

According to the theory, the vertices of this polytope should represent optimal specialist phenotypes in trait space. Thus, these archetypes are predicted to be significantly enriched in certain particular functions, some of which being either different or performed by different genes, and others shared among different archetypes. FDR-adjusted Fisher’s exact-tests on archetype defining gene lists clearly show that these genes are non-random genomic sub-samples, being statistically highly significantly enriched in particular functions. Some of the most significantly enriched functions were different among archetypes, although the third and fourth archetypes showed substantial overlap in broadly defined functions, but little overlap in the genes underlying these functions within each archetype (the overall percentage of positively and negatively correlated genes unique to either archetype in pairwise comparison was 72.5%). The fact that different archetypes are statistically significantly enriched in different functions and/or gene combinations rules out the possibility that the observed functional enrichment is simply reflecting the general “B-lymphocyte” phenotype and is rather consistent with each archetype representing distinct optimization solutions. Given the typical complete effacement of lymph node architecture and extensive infiltration by malignant B-lymphocytes in DLBCL, these specialized functional sub-categories are likely to reflect, at least partly, various B-cell malignant phenotypic strategies, although some contribution from other cell types from the TME such as dendritic cells, macrophages or T-lymphocytes cannot be completely excluded. Nevertheless, even in the unlikely possibility that the archetypes represent cell-type specializations rather than tumor-cell specializations, Pareto theory’s predictions (polyhedron structure of the data, functional enrichment at the vertices, shared/modular structure of the functions/genes characterizing the phenotypes) would remain confirmed, albeit at a different level of cellular differentiation. Indeed, this theory scales to different levels of biological organization and has been applied to test for phenotypic optimization in various kinds of biological entities from cells to different animal species (e.g., Tendler et al., 2015). Thus, this possibility would not change the general conclusion that the theory is valid and that the archetypes identified represent trade-offs in the functions defining those archetypes.

However, besides these different functional characteristics, archetypes also displayed significant proportions of shared genes. As predicted if archetypes result from optimization in the face of trade-offs, these shared elements were distributed in different combinations and proportions among different archetypes. This pattern is consistent with archetypes having to reconcile various functional constrains by shuffling certain genetic toolkits, turning on and off the expression of genes in a way that preserves certain core functions and functional combinations, at the expense of other less essential and potentially dispensable elements. Whether the archetypes identified by the ParTI approach represent irreversible commitment to certain phenotypic pathways or phenotype through which cells can cycle sequentially remains an open question. The former possibility could thus represent stages in malignant progression, while the latter would include the possibility that different archetype might represent temporary adaptations to transient intra or extra-cellular environmental circumstances. Our analysis also confirmed that the archetypes identified by the Pareto approach are biologically interpretable and can be used to generate hypotheses about possible mechanisms underlying the identified correlation patterns. Thus, taken together, these results broadly confirm the predictions of the Pareto optimality theory as applied to these transcriptomic data.

If trade-offs can be identified by the Pareto task inference approach, this could open the possibility to exploit these results to develop therapeutic strategies tailored to minimize the risk of resistance. Thus, the metabolic and immune evasion trade-offs suggested by the data may represent therapeutic opportunities that deserve further study. The first trade-off supports recent findings suggesting an important role for mitochondria, OXPHOS and ROS in tumorigenesis (Ghosh et al., 2020; Liu and Shi, 2020; Vasan et al., 2020), including in relation with the stress responses induced by increased ROS production and their impact on macromolecule synthesis (Jin et al., 2022). The second trade-off is in line with recent suggestions that in follicular lymphomas, loss of MHCII may be selectively acquired in cells that have accumulated immunogenic mutations in their idiotype sequences in order to avoid displaying Ig neoantigens to T-cells (Han et al., 2022b). In that study, MHCII expressing lymphoma cells were associated with a TME rich in a CD4^+^ T-cell population with a high cytotoxicity expression profile signature (CD4_CTL_), while the reverse was observed for cells expressing low levels of MHCII (Han et al., 2022b). The pattern revealed by ParTI is also consistent with earlier findings that MHC loss can occur through a partial plasmablastic phenotypic differentiation which could be associated with high levels of Ig production (Wilkinson et al., 2012). As mentioned previously, the retention of MHC associated with the expression of CXCL9/10 and CCL18/19 in archetype 3 might represent an alternative immune evasion strategy to avoid NK-cells detection (from the expression of MHC and the action of CXCL9/10 and CCL19) and promote T-cell tolerance from CCL18 (Korbecki et al., 2020; Cardoso et al., 2021; Seliger and Koehl, 2022). Uncovering the best way to exploit such trade-offs in energy production, protein synthesis and immune evasion, will require additional detailed studies focused at testing the effect of disrupting the function of specific archetype defining genes on both side of the trade-offs simultaneously.

## Data Availability

Publicly available datasets were analyzed in this study. This data can be found here: Genome Data Commons Data Portal at GDC (cancer.gov).

## Supplementary material

SUPPLEMENTARY FIGURE S1 Typical distribution of gene expression coefficients at archetype locations. Most genes have coefficients very close to zero (slope close to zero), with a small subset of genes having strongly negative or positive coefficients (slope close to one).

SUPPLEMENTARY FIGURE S2 Archetype defining genes selection method. The intersection of horizontal (slope close to zero) and vertical (slope close to one) lines was bisected at 45° and the mid-point of this 45° bisecting line was used as the cutoff coefficient value for archetype defining genes.
