# Supplementary figures and images for: Pareto task inference analysis reveals cellular trade-offs in diffuse large B-Cell lymphoma transcriptomic data
Source: Front Syst Biol. 2024 Mar 1;4:1346076. doi: 10.3389/fsysb.2024.1346076 (PMC12342016; doi:10.3389/fsysb.2024.1346076)

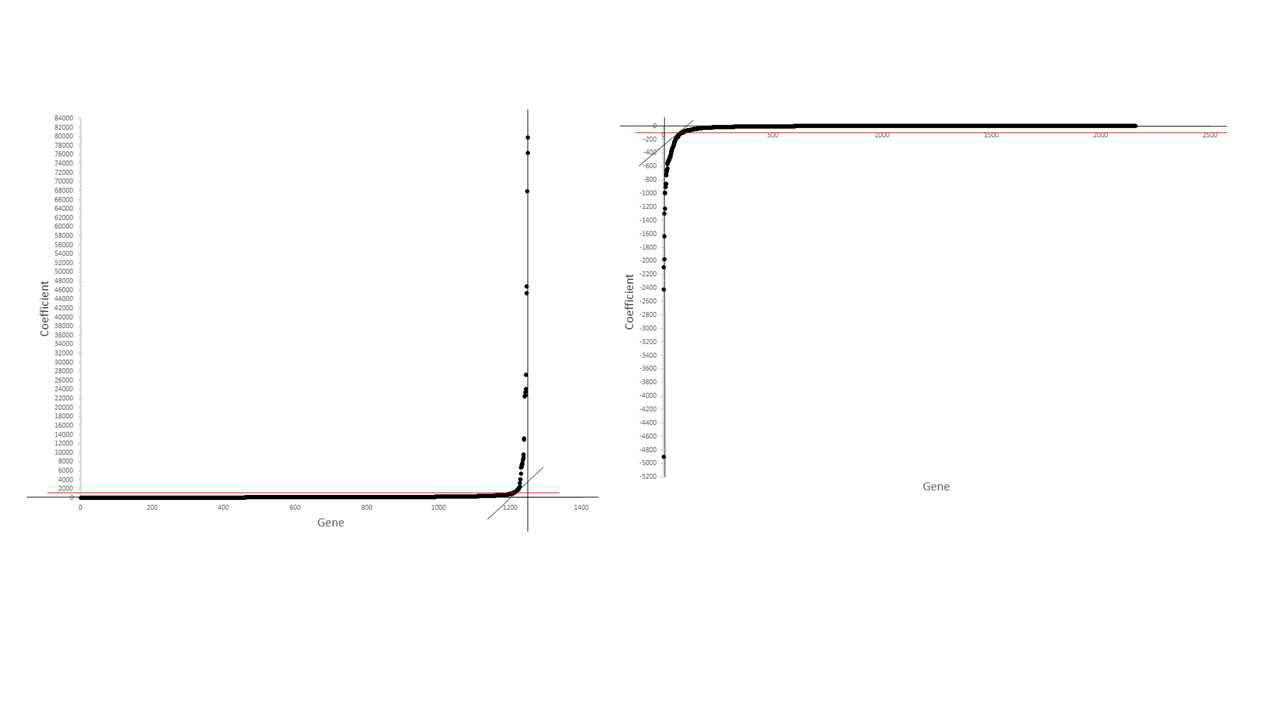

Supplement: Supplementary file 3 [file Image2.TIF]

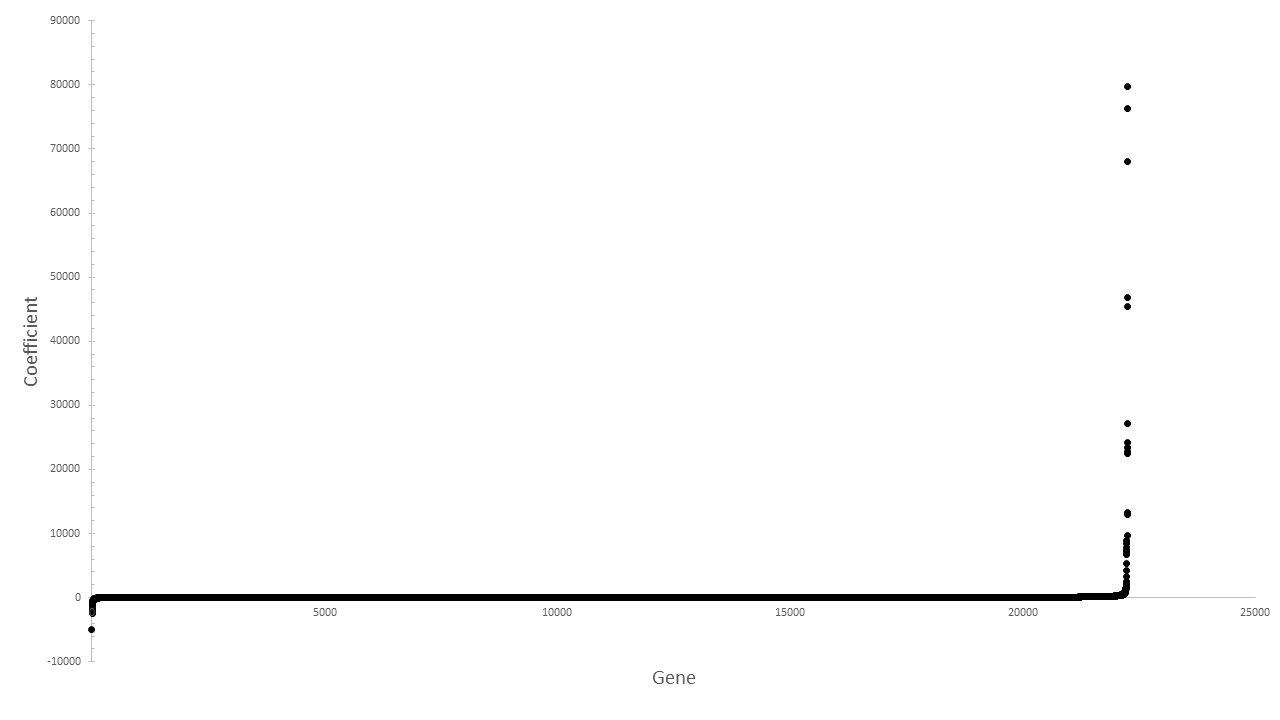

Supplement: Supplementary file 4 [file Image1.TIF]
