# Supplementary material for: Pareto task inference analysis reveals cellular trade-offs in diffuse large B-Cell lymphoma transcriptomic data
Source: Front Syst Biol. 2024 Mar 1;4:1346076. doi: 10.3389/fsysb.2024.1346076 (PMC12342016; doi:10.3389/fsysb.2024.1346076)
Supplement: Supplementary file 6 [file DataSheet1.docx]

## Data conversion from public data download to ParTI analysis (Bash and Rscript)

#___________________________________________________________________________

#___________________________________________________________________________

# Starting with a Data/ folder containing individual patient folders (TPM expression values for 481 cases from the NCICCR-DLBCL project, available at (GDC (cancer.gouv)), each with a *counts.tsv file

# Format for ParTI: a single file for gene expression values, with one patient per row and one gene per column (but no headers), gene symbols in a separate file

# Produce a file with gene symbols

cat Data/(any patient folder)/*counts.tsv | cut -f2 > Gene_symbols.txt

# Paste all TPM counts into a single file

less Gene_symbols.txt > TPM_nonunique_temp.txt

ls -d Data/* | while read line ;do

paste <(cat TPM_nonunique_temp.txt) <(cat ${line}/*counts.tsv |cut -f7) > temp.txt

mv temp.txt TPM_nonunique_temp.txt

done

# From TPM_nonunique_temp.txt, remove non-unique gene symbols

cat TPM_nonunique_temp.txt | sort -t$'\t' -k1,1 -u > TPM_selection.txt

# Transpose and remove gene symbols from this file

for i in {1..482} ;do

Echo ${i}

paste -s -d',' <(cat TPM_selection.txt | awk NR\>2 | grep -v tpm_unstranded | cut -f${i}) >> TPM_transposed.csv

done

cat TPM_transposed.csv | awk NR\>1 > TPM_noheader.csv

# Vector of gene symbols

cat TPM_selection.txt | awk NR\>2 | grep -v tpm_unstranded | cut -f1 > genesymbol_sel.csv

# Remove genes with expression variance lower than 1 (Rscript from here)

#_______________________________________________________________________

# Load TPM values and genes symbols in R

TPM=read.csv("TPM_noheader.csv",header=F)

sym=read.csv("genesymbol_sel.csv",header=F)

# Vector of variance per column

x=apply(TPM,2,var)

# Filter data and gene symbols based on variance

sym_sel=sym[x[]>1,]

TPM_sel=TPM[,x>1]

# Export

write.table(TPM_sel,"TPM_filtered.csv",row.names=FALSE, col.names=F, quote=FALSE, sep=",")

write.table(sym_sel,"genesymbol_filtered.csv",row.names=FALSE, col.names=F, quote=FALSE, sep=",")

#______________________________________________________________________

#ParTI code (matlab):

# Load the data into Matlab from a comma separated value (CSV) file

# The file is a purely numerical matrix, with patients as rows and genes as columns

geneExpression = dlmread('Directory/Data/"TPM_sel".csv', ',');

# Import discrete and continuous sample attributes, i.e. the clinical data on patients.

# Loading a file with clinical attributes, both discrete and continuous, in same order as expression data file (TPM_sel.csv).

[discrAttrNames, discrAttr] = ...

read_enriched_csv('Directory/Data/"discrclinicaldata".csv', char(9));

[contAttrNames, contAttr] = ...

read_enriched_csv('Directory/Data/"contclinicaldata".csv', char(9));

#run ParTI

# Uses the SDVMM algorithm (4), with up to 8 dimensions. Provides the discrete patient attributes, and ask ParTI to preliminary booleanize these attributes (0). Passes continuous patient attributes. Specifies that the enrichment analysis will be performed with a bin size of 5%.

[arc, arcOrig, pc, errs, pval] = ParTI(geneExpression, 4, 8, discrAttrNames, ...

discrAttr, 0, contAttrNames, contAttr, 0.05);

# Extracting archetype coefficients in original gene expression space from ParTI's output

# Export ParTI's output file "arcOrig" (a matrix of 4 archetypes X 22 250 coefficients) as csv file:

writematrix(arcOrig, coefficients.csv);

#____________________________________________________________________

#___________________________________________________________________________

## Data sorting after ParTI analysis (Rscript)

# Matches each coefficient to its corresponding gene symbol

# Sort coefficients in decreasing order

# Starting with the list of gene symbols and a file containing archetype coefficients from ParTI

gene=read.csv("genesymbol_filtered.csv", header=F)

coeff=read.csv("coefficients.csv", header=F)

#Combine into a dataframe

all=data.frame(gene[,1],t(coeff))

# Write one file per archetype, each with genes ordered by decreasing coefficient value

write.table(all[order(-all$X1),c(1,2)],"arch1.txt",row.names=FALSE, quote=FALSE,sep="\t")

write.table(all[order(-all$X2),c(1,3)],"arch2.txt",row.names=FALSE, quote=FALSE,sep="\t")

write.table(all[order(-all$X3),c(1,4)],"arch3.txt",row.names=FALSE, quote=FALSE,sep="\t")

write.table(all[order(-all$X4),c(1,5)],"arch4.txt",row.names=FALSE, quote=FALSE,sep="\t")
